# Supplementary material for: Construction and Evaluation of an Efficient Live Attenuated Salmonella Choleraesuis Vaccine and Its Ability as a Vaccine Carrier to Deliver Heterologous Antigens
Source: Vaccines (Basel). 2024 Feb 27;12(3):249. doi: 10.3390/vaccines12030249 (PMC10974600; doi:10.3390/vaccines12030249)

Supplementary Material

Supplementary Table S1. Primers used in this study

Primer ID	Sequence (5’-3’)
rpoS-F	ggtaacgcactgcgtggttacg
rpoS-R	ggtcacggtttcaccaaaagtg
crp-1F	gaacgtgagtccttcaacccac
crp-1R	cgccattctgacgggagcgcggttatcctctgt
crp-2F	aaccgcgctcccgtcagaatggcgcgttttatca
crp-2R	agcaccagcgtcagcccgg
fur-1F	cacctcgacgacatcctcaac
fur-1R	agcggaatctgtcctgttgcctaaacgattcacttc
fur-2F	tttagcaacaggacagattccgctaagtgtaaatctttc
fur-2R	tgctctgcagtagacggatgg
phop-1F	caagctggaagtaaaccgcgatc
phop-1R	caaatttattcacatctcttctcccttgtgttaacaataag
phop-2F	gagaagagatgtgaataaatttgctcgccattttctgc
phop-2R	ctcgctcaacagcgtgctgg
aroA-1F	gtgttcaaatggttgaaagcgcagg
aroA-1R	caacagaagacgaaactcaactctcaaaaaacagaaataaaaaccc
aroA-2F	gagagttgagtttcgtcttctgttgcgccagtcgac
aroA-2R	tgccagttgattctcgctgtcc

Supplementary Figure S1. The growth curves of strains under different culture conditions. (A) Growth curves of S. Choleraesuis wild type C3545, vaccine strain C500, mutant strain C5000 (C500 rpoS+) in LB broth. (B) Growth curves of S. Choleraesuis wild type C3545, vaccine strain C500, mutant strain C5000 (C500 rpoS+) in LB with 10 mM H2O2 broth. (C) Growth curves of S. Choleraesuis wild type C3545, vaccine strain C500, mutant strain C5000 (C500 rpoS+) in LB broth (pH=4.5). (D) Growth curves of S. Choleraesuis wild type C3545, vaccine strain C500, mutant strain C5000 (C500 rpoS+) in LB broth (pH=7.4).

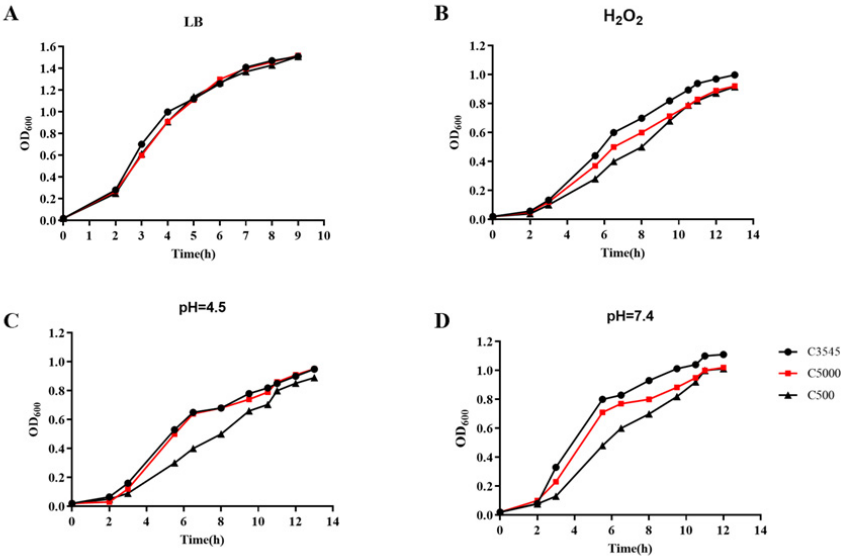

Supplementary Table S2. Virulence of C500 and C5000 (C500 rpoS+) in 7-week-old BALB/c mice. Virulence was expressed as the LD50 value (CFU) for the mice.

Strain	Description	LD_{50} (CFU)
C500	Attenuated vaccine strain	$>5.2*10^9$
C5000	C500 *rpoS*$^+$	$3.5*10^9$

Virulence expressed as the LD_{50} value (CFU) for the mice.

Supplementary Figure S2. Biological characteristics analysis of the strains.

Biological characteristics analysis of the strains. Growth curves of S. Choleraesuis wild type C3545, vaccine strain C500, mutant strains C5001 (C500 rpoS+ crp10), C5002 (C500 rpoS+ fur9), SC1 (C500 ΔpagL7 ΔpagP81::Plpp lpxE ΔlpxR9), SC2 (C500 rpoS+ Δcrp10 ΔpagL7 ΔpagP81::Plpp lpxE ΔlpxR9), and SC3 (C500 rpoS+ Δfur9 ΔpagL7 ΔpagP81::Plpp lpxE ΔlpxR9) in LB broth.

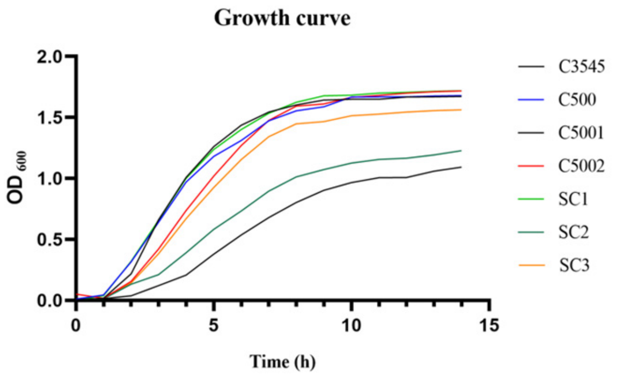

Supplementary Figure S3. Histopathological analysis.

Histopathological analysis. Mice were inoculated with Salmonella spp strains, and the effect on the organs was investigated and compared with the organ samples of mice receiving BSG as a placebo. Histopathological changes in the spleen collected at 6 days were studied.

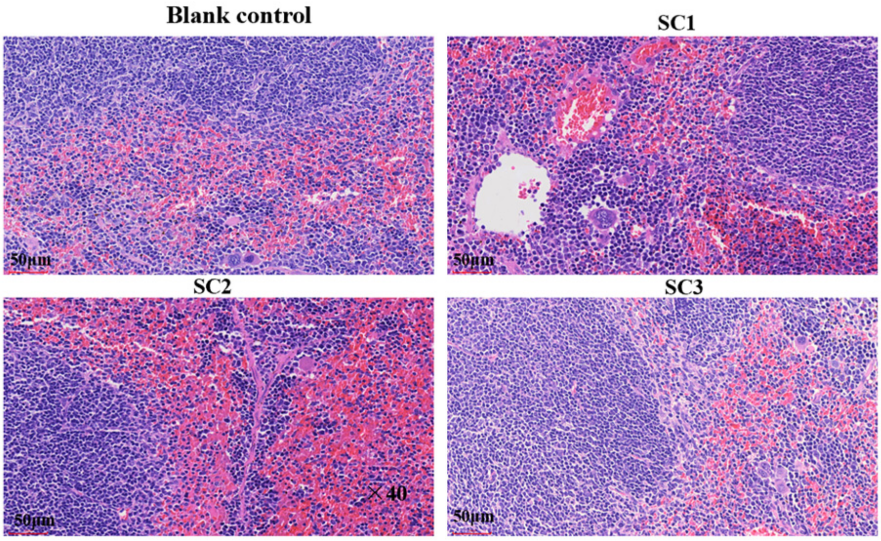

Supplementary Figure S4: The whole blot (uncropped blots) of Figure 7A. From left to right, lane 1 is strains SC3 Δ*asd* and lane 2 is strains SC3 Δ*asd* (pSW-O9) pellets were separated by SDS-PAGE and transferred to nitrocellulose membranes for Western Blot analysis using rabbit anti-*Salmonella* O7 serum. **Other lanes were not relevant to this study. Western Blot analysis of O-antigen samples does not require molecular weight markers.**

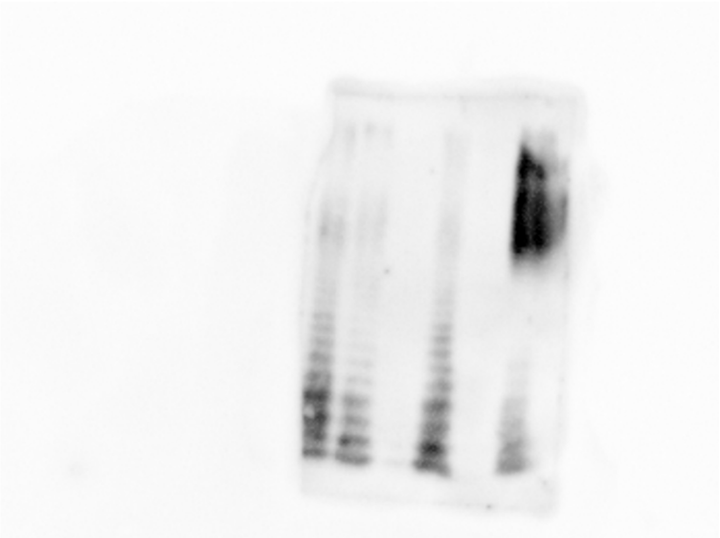

Supplementary Figure S5: The whole blot (uncropped blots) of Figure 7B. From left to right, lane 1 is strains SC3 Δ*asd*, lane 2 and 3 is strains SC3 Δ*asd* (pSW-O9) pellets were separated by SDS-PAGE and transferred to nitrocellulose membranes for Western Blot analysis using rabbit anti-*E. coli* O9 serum. **The remaining two lanes were identical to lanes 2 and 3, with the volume of samples halved. Western Blot analysis of O-antigen samples does not require molecular weight markers.**

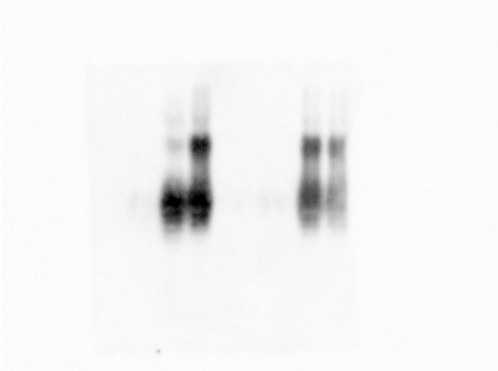

Supplementary Figure S6: The whole blot (uncropped blots) of Figure 7C.

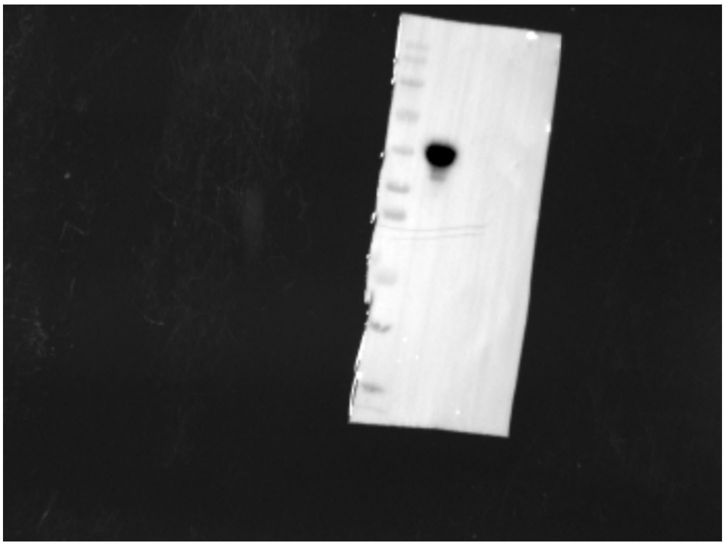

Supplement: Supplementary file 1 [file vaccines-12-00249-s001.zip › vaccines-2796551-supplementary.pdf]
